# Effect of Endothelin-1 on Baroreflexes and the Cardiovascular Action of Clonidine in Conscious Rabbits

**DOI:** 10.3389/fphys.2016.00321

**Published:** 2016-07-28

**Authors:** Kyungjoon Lim, Maarten van den Buuse, Geoffrey A. Head

**Affiliations:** ^1^Neuropharmacology Laboratory, Baker IDI Heart and Diabetes Research InstituteMelbourne, VIC, Australia; ^2^School of Psychology and Public Health, La Trobe UniversityMelbourne, VIC, Australia; ^3^Department of Pharmacology, University of MelbourneMelbourne, VIC, Australia; ^4^Department of Pharmacology, Monash UniversityClayton, VIC, Australia

**Keywords:** endothelin, clonidine, blood pressure, heart rate, intracisternal injection, conscious rabbits

## Abstract

We studied the influence of pretreatment with endothelin–1 on cardiac baroreflexes and on the effect of clonidine on blood pressure and heart rate. In order to avoid the complication of the direct vasoconstrictor effects of endothelin-1, initial dose-response studies in animals treated with a ganglion blocker were performed. Intravenous administration of 50, 200, and 1200 ng/kg of endothelin-1 produced biphasic changes in blood pressure, consisting of an immediate depressor response, followed by a long lasting and dose-dependent pressor effect (peak response 3 ± 1, 9 ± 3, and 33 ± 5 mmHg, respectively). Thus, the 50 ng/kg dose of endothelin-1 was used in subsequent studies. Conscious rabbits were pretreated on separate days with endothelin-1, either intravenously (50 ng/kg) or intracisternally (10 and 50 ng/kg), or with vehicle. The animals then received an intravenous dose (20 μg/kg) or an intracisternal dose (1 μg/kg) of clonidine and the effects on blood pressure and heart rate were measured. In vehicle-treated rabbits, the intravenous administration of clonidine induced a significant decrease in blood pressure and heart rate (15 min after injection: −15.7 ± 4.7 mmHg and −33 ± 4 b/min, respectively). Similarly, the intracisternal injection of clonidine lowered blood pressure (−16.0 ± 2.5 mmHg), but produced a less pronounced bradycardia (−18 ± 4 b/min). Endothelin pretreatment, either 50 ng/kg centrally or peripherally, had no significant effect on the hypotension or bradycardia produced either by central or peripheral injection of clonidine. At this dose, endothelin by itself did not produce significant changes in blood pressure or heart rate. There was a reduction of the gain of the baroreceptor-heart rate reflex with intracisternal endothelin-1. These results suggest that central _2_–adrenoceptor mechanisms involved in clonidine-induced hypotension and bradycardia do not appear to be influenced by activation of endothelin receptors.

## Introduction

Endothelin, an endothelium-derived 21 amino–acid peptide, is a potent vasoconstrictor which was isolated by Yanagisawa et al. (1988). Three isoforms of endothelin have been characterized: endothelin-1, endothelin-2, and endothelin-3, which differ in their distribution in the body and in their effects (Inoue et al., 1989; Sokolovsky, 1992). Intravenous injection of endothelins in rats evoked a characteristic biphasic effect on blood pressure, consisting of an early hypotension followed by a longlasting increase in blood pressure (Yanagisawa et al., 1988). Local production of endothelins in the vascular wall may play an important role in the regulation of blood pressure and haemodynamic flow and could be involved in the pathogenesis of cardiovascular disease (Sokolovsky, 1992; Huggins et al., 1993; Sandoo et al., 2010).

In addition to the systemic circulation, endothelin production and endothelin receptors have been demonstrated in the central nervous system. The central administration of endothelins modulates endocrine and cardiovascular regulation and behavior (van den Buuse, 1997). For example, endothelins were found to be involved in the regulation of vasopressin release (Yoshizawa et al., 1990; Yu et al., 2001) and baroreflex sensitivity (Itoh and van den Buuse, 1991; Abukar et al., 2016). Furthermore, injections of endothelins intracisternally or locally into the rostral ventrolateral medulla produced marked changes in blood pressure (Kuwaki et al., 1991; Mosqueda-Garcia et al., 1992; Kumada et al., 2003; Lu et al., 2005).

Clonidine is a clinically used centrally-acting antihypertensive agent with agonist activity at α_2_–adrenoceptors and additional actions at the imidazoline-preferring receptors (Kobinger, 1978; Ernsberger et al., 1987). It lowers blood pressure through inhibition of sympathetic vasomotor tone and a reduction of cardiac output (Badoer et al., 1983). The site of action of clonidine is most likely in the medulla oblongata (Punnen et al., 1987; Gatti et al., 1988).

Studies by Gulati and co-workers have led to the suggestion of an interaction between the effects of endothelin and clonidine in anaesthetized rats (Gulati, 1992; Gulati and Srimal, 1993). Intravenous administration of subpressor doses of endothelin blocked the hypotension and bradycardia produced by clonidine but did not inhibit the initial hypertensive response. The authors suggested that endothelin crossed the blood-brain barrier to block the central hypotensive actions of clonidine, and that central and peripheral α2–adrenoceptors therefore may be differentially influenced by endothelin. The pharmacological interaction between endothelin and clonidine suggested in these studies is unexpected as these compounds clearly interact at distinct and dissimilar receptors. The authors examined this question in urethane-anaesthetized rats and found that endothelin antagonized the effects of clonidine and that endothelin antagonists, such as the ETA/B antagonist, TAK-044, and the ETA antagonist, BMS-182874, potentiated the effects of clonidine (Lavhale et al., 2010). They also found that clonidine produced an increase in ETA receptor expression in the brain and abdominal aorta and concluded that endothelin enhances the responsiveness of vascular adrenoceptors to the constrictor effect of clonidine and ET antagonists potentiate the hypotensive effect of clonidine (Lavhale et al., 2010). One possibility was that this interaction was a functional rather than a pharmacological one. Thus, it is important to examine the interaction of endothelin and clonidine using doses of endothelin that have minimal direct vascular constrictor effects.

The principle aim of the present study was to investigate the central and peripheral cardiovascular effects of endothelin and its interaction with clonidine using a conscious animal preparation. In this study we used rabbits since the haemodynamic and cardiovascular reflex effects of clonidine have been very well characterized in this species (Badoer et al., 1983; Head and Burke, 1991). We examined both the effects of central (intracisternal) and systemic (intravenous) treatment with endothelin on blood pressure and heart rate and the interaction of pretreatment with endothelin on blood pressure responses to intravenous and intracisternal administration of clonidine. We also examined the effects of central doses of endothelin on baroreceptor heart rate reflexes.

## Materials and methods

### Animals, operations, and measurements

All experiments and surgical procedures were approved by the Baker Medical Research Institute/Alfred Hospital Animal Ethics Committee and were in accordance with the guidelines of the National Health and Medical Research Council of Australia. The experiments were performed in 6 (dose-response studies), 7 (interaction studies), and 9 (baroreflex studies) conscious male and female rabbits of mixed breed with a body weight ranging between 2.4 and 3.0 kg.

For the interaction studies, during a preliminary operation the animals were anaesthetized with halothane (*Fluothane*, ICI Australia) after induction with intravenously administered propofol (*Diprivan*, ICI Pharmaceuticals, England, 10 mg/kg). A vinyl catheter of 0.28 mm internal diameter and 0.61 mm outer diameter (SV10, Dural Plastics, Australia) was inserted 8 mm through the atlanto–occipital membrane such that the tip lay in the fourth ventricle (Head et al., 1983). The first experiment was performed approximately 1 week after surgery.

For the barororeflex studies under similar anaesthesia, small silastic aortic and vena caval balloons were implanted in the chest of rabbits in two separate operations with at least 2 weeks recovery in between (Korner et al., 1979).

On the day of the experiment the central ear artery and marginal ear vein were catheterized transcutaneously with a 25 mm, 22 gauge Teflon catheter (*Jelco*, intravenous catheter placement unit, Johnson and Johnson Medical, Australia) and a 23 gauge stainless steel needle catheter, respectively, under local anaesthesia with 1% prilocaine (*Citanest*, Astra Pharmaceuticals, Australia). The end of the intracisternal catheter was retrieved, using local anaesthesia, from under the skin at the back of the neck.

Mean arterial pressure (MAP) and heart rate were recorded on a polygraph (Grass, model 7D). For the MAP measurements, Statham P23D transducers were used, while heart rate was measured using a Baker Medical Research Institute rate meter which was triggered by the pulsatile arterial pressure signal. The MAP and heart rate signals were digitized with an analog-to-digital conversion card (Metrabyte DAS-8) and PC compatible computer. Software developed at the Baker Medical Research Institute was used to average the data over 20 s periods for analysis. At least 1 h recovery from the minor operative procedures was allowed before the experiment was begun.

### Experimental protocols

Preliminary experiments were performed to investigate the pressor action of endothelin-1 in conscious rabbits and to assess the biological activity of the endothelin-1 used in our studies. Six rabbits were treated intravenously with a ganglionic blocker (mecamylamine, 4 mg/kg, or pentolinium, 5 mg/kg) and injected intravenously with different doses of endothelin-1 (Auspep, Australia, 50, 200, and 1200 ng/kg) with 10-15 min intervals between injections.

In the main series of experiments, in which we investigated the possible interaction of endothelin–1 and clonidine HCl (Catapres, Boehringer Ingelheim), each of the 7 rabbits was studied on six separate occasions, with 2–3 days between experiments. The order of treatments over the six separate experiments was determined according to a Latin square design. These consisted of (a) intravenous (*n* = 3) or intracisternal (*n* = 4) pretreatment with vehicle (Ringers Injection Solution) followed by intravenous injection of 20 μg/kg clonidine, (b) intravenous pretreatment with 50 ng/kg of endothelin-1 followed by intravenous clonidine (*n* = 7), (c) intracisternal pretreatment with 10 ng/kg of endothelin-1 followed by intravenous clonidine (*n* = 7), (d) intracisternal pretreatment with 50 ng/kg of endothelin-1 followed by intravenous clonidine (*n* = 7), (e) intracisternal injection of vehicle followed by intracisternal injection of 1 μg/kg of clonidine (*n* = 7), (f) intracisternal injection of 10 ng/kg of endothelin-1 followed by intracisternal injection of 1 μg/kg of clonidine (*n* = 7). In all cases, after an initial 20 min control period was recorded, the rabbits received their pretreatment. A 5 min post-treatment control period was then recorded 30 min after this first injection to indicate any effect on resting MAP or heart rate followed by the intravenous or intracisternal injection of clonidine. Changes to MAP and heart rate were then observed for the next 60 min. Two-minute digitized recordings were taken at regular intervals after clonidine administration. The effect of clonidine was taken during the maximum fall in MAP at 15 min post-injection for subsequent statistical analysis.

For the baroreflex cuves, 20–30 inflations of the venous cuff were performed to reduce venous return and hence arterial pressure or of the aortic cuff to increase aortic and carotid pressure before and after vehicle or endothelin-1 (2 ng/kg, 10 ng/kg). Each cuff was held for 30 s at a steady state pressure. Aortic and venous cuffs were alternated to avoid baroreceptor resetting. The final values (at the end of 30 s) for MAP and HR were averaged over 2 s intervals and fitted to a sigmoid logistic function to produce MAP-HR curves. A non-linear regression program utilized the Marquardt-Levenberg method to fit a five parameter logistic equation as described previously (Ricketts and Head, 1999). Parameters included the lower plateau, which was the minimum HR, the range between the lower plateau and upper plateau (which was a calculated maximum activation) and the median blood pressure at half the reflex range (BP50). Two curvature parameters were used which allowed for a non-symmetrical fit of the data. The average range-dependent slope between the two inflection points of the first derivative curve (gain) was calculated as the average of the curvature parameters multiplied by the range and divided by 4.562 (Ricketts and Head, 1999).

Both endothelin-1 and clonidine were dissolved in sterile Ringers injection solution. The intravenous injection volume was 1 ml followed by a 400 μl flush of Ringers, whereas the intracisternal injection volume was 25 μl followed by a 20 μl flush.

### Statistical analysis

All values in the text are expressed as mean ± standard error of the mean (SEM) or mean difference ± standard error of the difference (SED) as appropriate. The pressor response to intravenously administered endothelin-1 was analyzed with one-way analysis of variance. The effect of clonidine in vehicle-treated and endothelin-treated rabbits was combined in a single repeated-measures two–factor analysis of variance where the factors of “drug” and “animal” were removed from the total sums of squares to give the residual sums of squares. The latter was used to calculate the average “within animal” SEM (Snedecor and Cochran, 1980). The effect of endothelin compared to vehicle was determined by three sets of non-orthogonal contrasts. Thus, the Bonferonni adjustment was made to correct for multiple comparisons (Wallenstein et al., 1980).

## Results

### Dose-response studies

**Figure 1:**
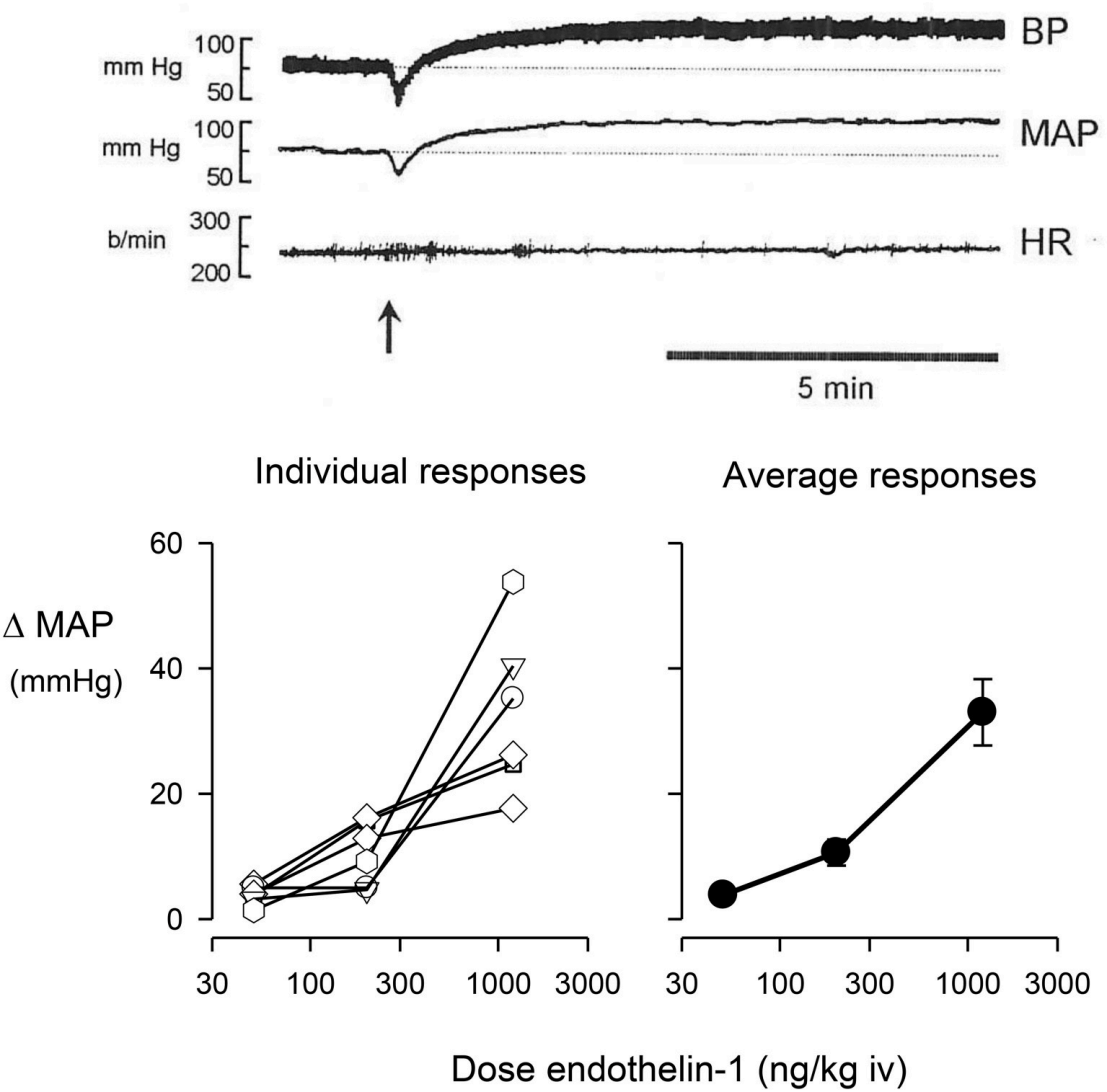
**Change in mean arterial pressure (MAP) induced by the intravenous (iv) injection of endothelin-1**. The **top panel** shows a typical trace of pulsatile blood pressure, mean blood pressure, and heart rate of a conscious, ganglion blocked rabbit, before and after injection of 1200 ng/kg of endothelin-1 shown at arrow. The **bottom left panel** shows individual maximal pressor responses of six conscious rabbits to doses of 50, 200, or 1200 ng/kg of endothelin-1, while the **bottom right panel** shows the average responses of these animals.

Ganglionic blockade in conscious rabbits caused a moderate fall in blood pressure (−11.7 ± 5.1 mmHg) and a tachycardia (66 ± 10 b/min). The intravenous injection of endothelin-1 in ganglion-blocked rabbits caused an immediate and dose-dependent decrease in blood pressure, rapidly followed by a long-lasting pressor response (Figure 1). Injection of endothelin-1 at the lowest dose of 50 ng/kg caused a small increase in MAP of +3.0 ± 0.8 mmHg. At a dose of 200 ng/kg of endothelin-1, the maximal change in blood pressure was +9.3 ± 2.9 mmHg, whereas the highest dose of 1200 ng/kg of endothelin-1 increased blood pressure by +33.0 ± 5.3 mmHg (Figure 1). In these ganglion-blocked rabbits, there were very minimal effects of endothelin-1 treatment on heart rate (not shown). Thus, there was only a minimal pressor effect of the dose of 50 ng/kg, which was subsequently used in our experiments to examine the interaction of endothelin with the effects of clonidine.

### Effect of endothelin treatment on the action of clonidine

During the 3-week experimental period in which a total of 6 interaction experiments were performed, the average of body weight, MAP and heart rate of these rabbits was 2.6 ± 0.1 kg, 74.5 ± 0.8 mmHg, and 153 ± 2 b/min, respectively. The rabbits remained in good health and there were no significant changes to any of these variables during this time. The final average value in each case was within 4% of the initial value.

**Figure 2:**
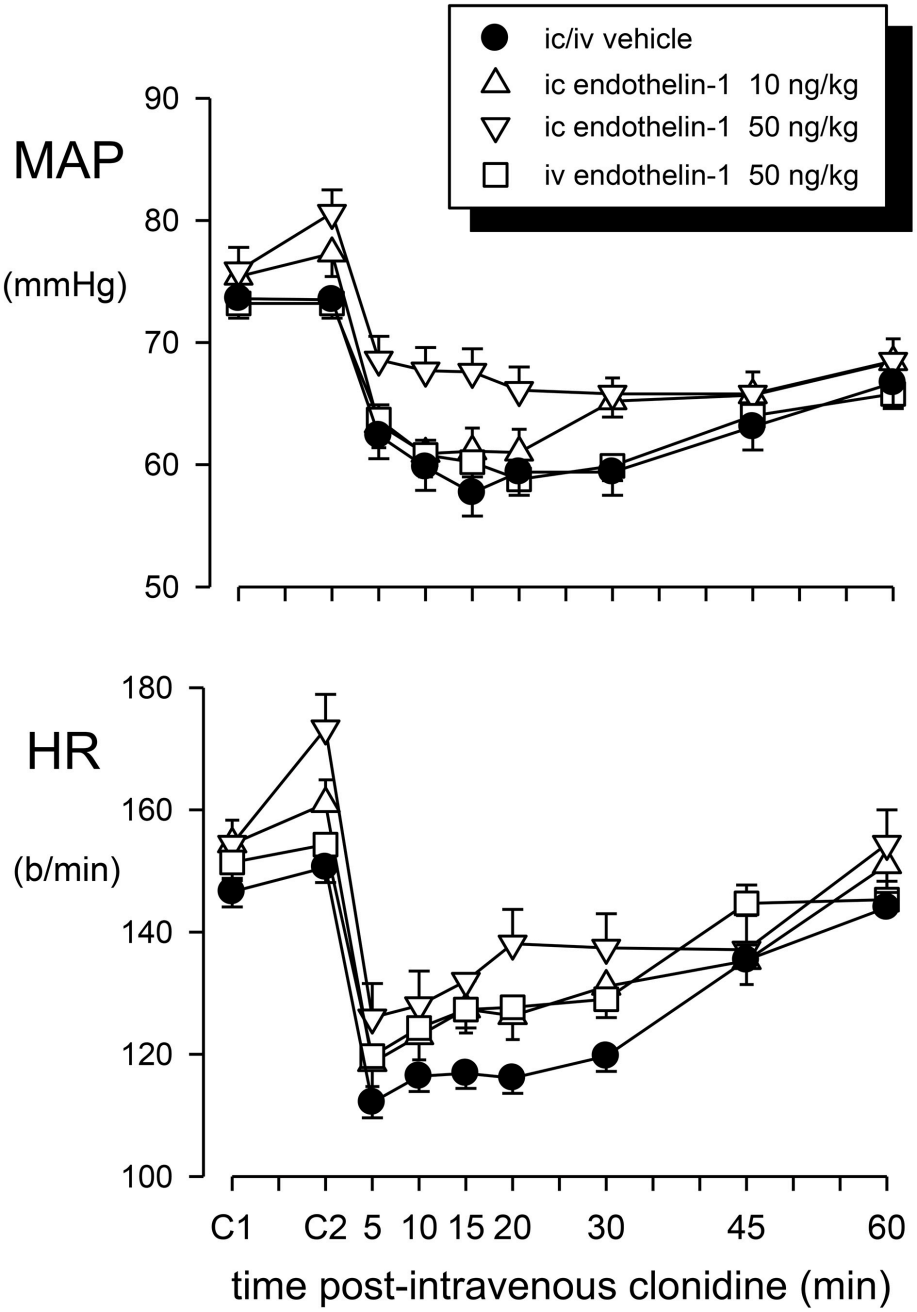
**Mean arterial pressure (MAP) and heart rate (HR) responses to intravenous (iv) injection of clonidine (20 μg/kg) over a 60 min time course following vehicle pretreatment (closed circles) or pretreatment with endothelin–1: 50 ng/kg intravenously (squares), 10 ng/kg intracisternally (ic) (triangle up) or 50 ng/kg intracisternally (triangle down)**. C1 indicates initial basal values and C2 indicates MAP and heart rate 30 min after pretreatment injection. Error bars are ± 1 SEM from analysis of variance and indicate variation between animals.

**Figure 3:**
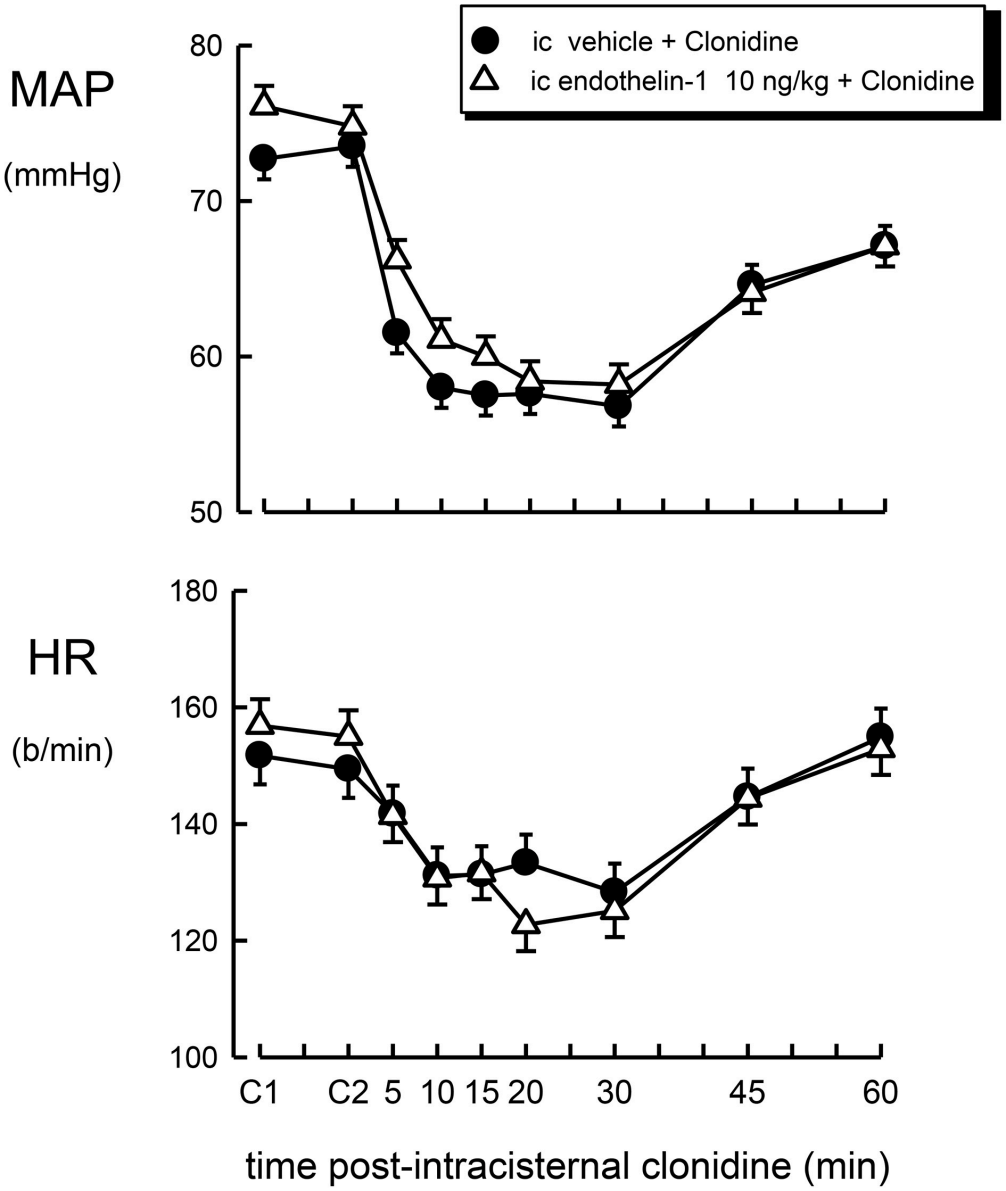
**Mean arterial pressure (MAP) and heart rate (HR) responses to intracisternal (ic) injection of clonidine (1 μg/kg) over a 60 min time course following intracisternal pretreatment with vehicle (filled circle) or endothelin–1 (10 ng/kg, open triangle)**. C1 indicates initial basal values and C2 indicates MAP and heart rate 30 min after pretreatment injection. Error bars, as for Figure 1.

Intravenous administration of 50 ng/kg of endothelin-1 did not significantly change MAP or heart rate from the initial control value in these rabbits (0.0 ± 1.1 mmHg and +2.9 ± 4.5 b/min, respectively; Figure 2). However, the same dose given intracisternally produced variable results. A large pressor (+26 mmHg at 30 min) and tachycardic response (+88 b/min) was observed in one of the seven rabbits, a moderate increase in MAP and heart rate was observed in two other animals, but there was little change in the other four animals. Thus, the overall average change in MAP (+4.7 ± 4.4 mmHg) and heart rate (+18.9 ± 11.9 b/min) was not significant (Figure 2). The lower dose of endothelin-1 (10 ng/kg) given intracisternally had no significant effect on basal MAP and heart rate (+1.9 ± 0.9 mmHg and +6.6 ± 4.1 b/min, respectively; Figures 2, 3).

Pretreatment with an intravenous or intracisternal injection of Ringers solution (vehicle) prior to systemic administration of clonidine had no effect on the basal MAP or heart rate (Figures 2, 3).

The effect of intravenous treatment with clonidine on blood pressure and heart rate in vehicle- or endothelin-1-pretreated rabbits is shown in Figure 2 and in Table 1. In vehicle-pretreated rabbits, intravenous injection of clonidine induced a decrease in blood pressure and heart rate by −15.7 ± 4.7 mmHg and −33 ± 4 b/min, respectively, at 15 min post-administration (*P* < 0.02 and *P* < 0.001, respectively, *n* = 7). A similar hypotension and bradycardia was induced by clonidine in rabbits pretreated intravenously with 50 ng/kg of endothelin-1 (*F*_1, 30_ between groups = 0.5 and 0.7, respectively, *P* > 0.05). Similarly, intracisternal pretreatment with 10 or 50 ng/kg of endothelin-1 did not alter the responses to intravenously administered clonidine (*F*_1, 30_ between groups = 0.1 and 0.3, respectively, *P* > 0.05). Thus, none of the depressor or bradycardic responses to clonidine administration following endothelin-1 pretreatment were significantly different to those observed in the vehicle-treated rabbits (Table 1).

**Table 1:** **Changes in mean arterial pressure (MAP) and heart rate (HR) induced 15 min following clonidine administration in vehicle or endothelin-1-pretreated rabbits**.

**Pretreatment**	**Dose (ng/kg)**	**Treatment**	**Dose (μg/kg)**	**Change in MAP (mmHg)**	**Change in HR (b/min)**
Vehicle	ic/iv	Clonidine	iv 20	−15.7 ± 4.7	−33 ± 4
Endothelin-1	iv 50	Clonidine	iv 20	−13.0 ± 1.2	−27 ± 6
Endothelin-1	ic 10	Clonidine	iv 20	−16.2 ± 3.4	−34 ± 8
Endothelin-1	ic 50	Clonidine	iv 20	−13.0 ± 2.3	−41 ± 8
Vehicle	ic	Clonidine	ic 1	−16.0 ± 2.5	−18 ± 4
Endothelin-1	ic 10	Clonidine	ic 1	−14.8 ± 2.0	−25 ± 4

Values are mean difference ± SED. ic, intracisternal; iv, intravenous.

The effect of intracisternal treatment with clonidine on blood pressure and heart rate in rabbits which were centrally pretreated with vehicle or endothelin-1 is shown in Figure 3. Intracisternal injection of clonidine lowered blood pressure in vehicle-treated rabbits by −16.0 ± 2.5 mmHg (*P* < 0.001), but produced a less pronounced bradycardia when compared to intravenous treatment (−18 ± 4 b/min, *P* < 0.005). Central pretreatment with 10 ng/kg of endothelin-1 had no significant effect on the hypotension or bradycardia produced by centrally injected clonidine (Table 1, Figure 2, *F*_1, 30_ between groups = 0.3 and 3.6, respectively, *P* > 0.05).

### Effect of ET-1 treatment on baroreflex curves

The central injection of 10 ng/kg of endothelin-1 in this group of conscious rabbits produced a prominent but short-lasting pressor (15–20 mmHg) and tachycardic (30–40 b/min) response. Furthermore, this dose of endothelin-1 produced a marked and significant reduction of the baroreceptor reflex gain (−15.1 ± 2.2 vs. −9.9 ± 1.4 b/min/mmHg, *P* = 0.003), which was due to a decrease in the curvature coefficient (Table 2). Neither heart rate range nor any of the other baroreflex parameters were altered. Central injection of vehicle did not affect basal MAP or heart rate. In contrast, in these rabbits we observed a significant increase in baroreflex gain (−11.0 ± 0.9 vs. −14.7 ± 2.3 b/min/mmHg), which could indicate a time-related effect on reflex sensitivity (Table 2). No other baroreflex parameters were altered by vehicle treatment. The central injection of 2 ng/kg of endothelin-1 did not cause any change in basal MAP, heart rate, heart rate range or any of the other baroreflex parameters but tended to cause a slight increase in baroreflex gain, similar to the effect seen after vehicle treatment.

**Table 2:** **Changes in barorflex paramaters before (control) and after administration of vehicle or endothelin −1**.

**Parameter**	**Control**	**Vehicle**	***P***	**Control**	**ET1 2 ng/kg IC**	***P***	**Control**	**ET1 10 ng/kg IC**	***P***
Resting MAP (mmHg)	87.6 ± 1.6	84.3 ± 1.4	0.15	91.6 ± 1.5	87.7 ± 1.9	0.09	90.4 ± 2.9	90.9 ± 2.3	0.819
Resting HR (b/min)	187 ± 4	186 ± 4	0.82	200 ± 8	204 ± 7	0.48	198 ± 9	205 ± 8	0.273
Lower plateau (b/min)	122 ± 6	121 ± 6	0.82	125 ± 7	125 ± 8	0.96	125 ± 7	121 ± 7	0.543
Range (b/min)	202 ± 14	205 ± 15	0.86	213 ± 8	215 ± 5	0.85	206 ± 12	220 ± 11	0.261
Upper plateau (b/min)	308 ± 14	309 ± 13	0.95	338 ± 3	340 ± 10	0.86	330 ± 10	340 ± 9	0.385
Curvature	0.26 ± 0.03	0.35 ± 0.07	0.06	0.25 ± 0.03	0.29 ± 0.03	0.38	0.35 ± 0.06	0.21 ± 0.04	0.006
BP50 (mmHg)	84.7 ± 1	82 ± 1.2	0.17	88.7 ± 1.5	85.6 ± 2.2	0.12	87.7 ± 2.6	88.1 ± 2.1	0.830
GAIN (b/min/mmHg)	−11 ± −1	−14.7 ± −2.3	0.03	−11.6 ± −1.5	−13.6 ± 1.3	0.22	−15.1 ± 2.2	−9.9 ± −1.4	0.003
*n*	6			5			6		

## Discussion

The present study demonstrates that intravenous injection of endothelin-1 in conscious rabbits induces a dose-dependent pressor response, similar to that described in rats and other species (Yanagisawa et al., 1988; Sokolovsky, 1992; Huggins et al., 1993). At doses that caused minimal vasoconstrictor effects or changes in blood pressure, neither systemic nor central pretreatment with endothelin–1 caused any significant effect on the cardiovascular responses to systemically or centrally administered clonidine in conscious rabbits. The doses of endothelin we used were the maximum that could be used to investigate a specific, pharmacological antagonism. Our results do not support the view expressed in the studies of Gulati (1992) and Gulati and Srimal (1993) that there is a central and peripheral interaction between the effects of clonidine and endothelin. It was suggested by these authors that endothelin antagonized the clonidine-induced hypotension and bradycardia but potentiated the initial hypertensive response induced by intravenously administered clonidine. This effect was longlasting, since the hypotension and bradycardia produced by clonidine were still attenuated 4 h after treatment with endothelin-1 (Gulati, 1992). Furthermore, after intracerebroventricular injection or topical application on the ventral surface of the medulla oblongata, endothelin-1 caused a retardation of the effects of similarly administered clonidine (Gulati, 1992; Gulati and Srimal, 1993). It is worth noting that the intracerebroventricular injection of angiotensin-II also caused a complete inhibition of the hypotension and bradycardia induced by either intravenous or intracerebroventricular injection of clonidine (Gulati and Srimal, 1993). This would indicate that this is not a specific interaction and can occur with other, non-endothelin peptides. The interaction between clonidine and endothelin was also examined by Lim et al. using urethane anaesthetized rabbits where they found that pretreatment increased the hypotension when given intravenously but not when given intracisternally. However, the basal pressures were not given but sample traces suggest that MAP was 130 mmHg which is twice that of a normal conscious rabbit (Lim et al., 1998).

It is well-established that the biphasic response to clonidine involves both peripheral and central α-adrenoceptors. The initial rise in pressure is due to the excitation of peripheral receptors while the dominating hypotensive action of clonidine is due to a central mechanism in which central α-adrenoceptors play an important role (Kobinger, 1978; Van Zwieten and Timmermans, 1979). There is evidence to suggest that endothelins may modulate systemic α-adrenoceptor-mediated sympathetic nervous system responses (Wiklund et al., 1989; Tabuchi et al., 1990; Ieda et al., 2004) and also central catecholamine release (Koizumi et al., 1992). In the former studies, the addition of endothelins reduced noradrenaline release from sympathetic nerve endings, but enhanced postjunctional α-adrenoceptor responses. Gulati (1992) concluded that, considering the differential effect of endothelins on the cardiovascular responses to clonidine, systemic and central α_2_-adrenoceptors involved in the action of clonidine are different. It is important to note that it was suggested that endothelin-1 would cross the blood-brain barrier in order to exert its effect on central α_2_-adrenoceptors. Such a mechanism is difficult to accept as Koseki et al. (1989) failed to find any central labeling after the systemic injection of radiolabelled endothelin and suggested that little, if any, endothelin could enter the brain from the systemic circulation. It cannot be excluded, however, that systemic endothelins affect central mechanisms through an action on circumventricular organs. Indeed, in regions such as the area postrema and subfornical organ, intravenous injection of low doses of endothelin caused cellular excitation (Ferguson and Smith, 1991; Wall et al., 1992). It should be stressed that the primary site of action of clonidine on blood pressure, heart rate, and sympathetic nervous system activity appears to be in the rostral ventrolateral medulla (Punnen et al., 1987; Gatti et al., 1988). The presence of endothelin receptors has been demonstrated in the ventrolateral medulla (Kohzuki et al., 1991; van den Buuse and Itoh, 1993) and, while intravenously injected endothelin is not likely to reach this site, intracerebroventricular (Gulati and Srimal, 1993) or intracisternal injection (present study) of endothelin-1 would be able to reach the ventrolateral medulla. However, we found no evidence of an interaction of endothelin with clonidine.

**Figure 4:**
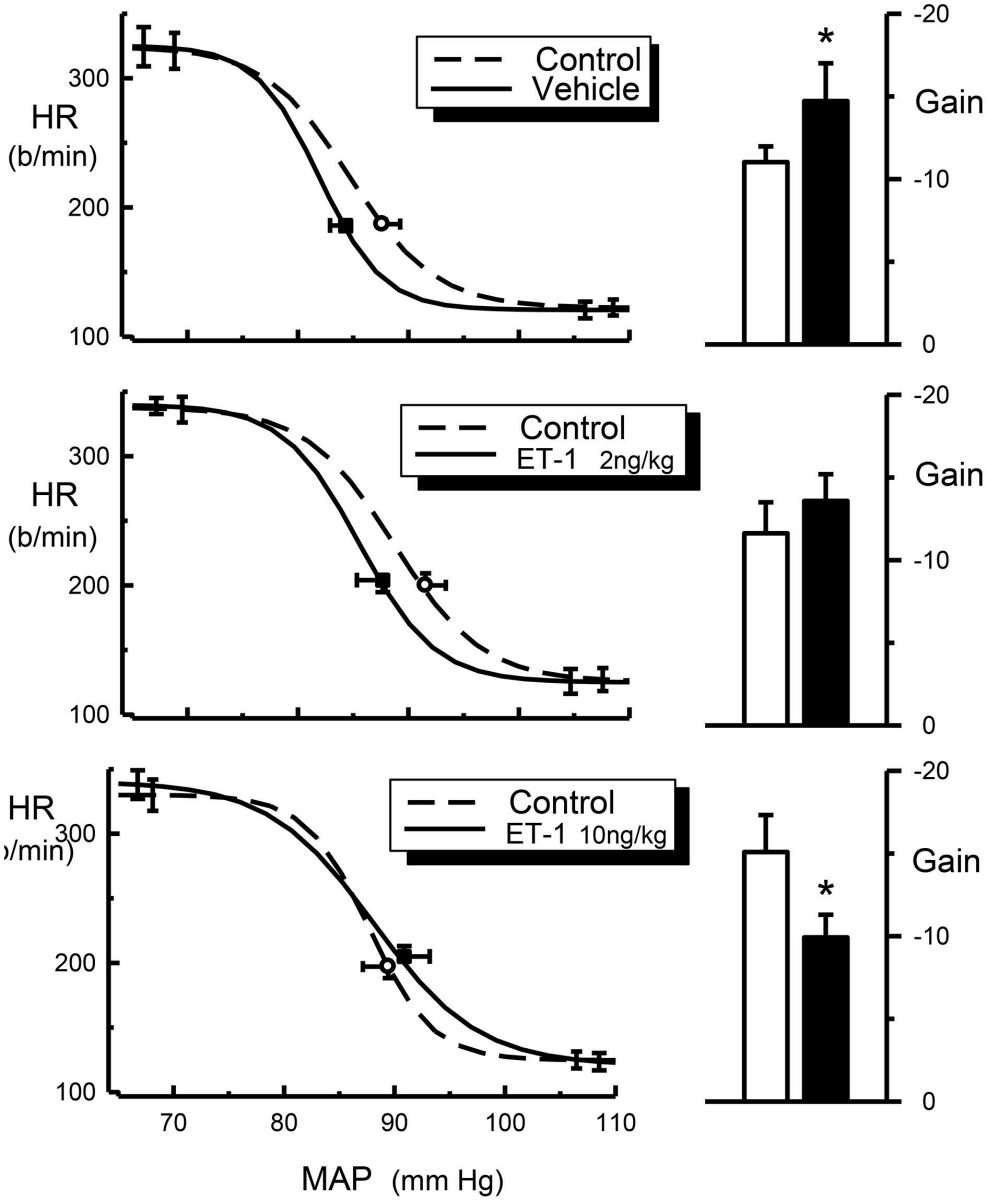
**Average HR baroreflex curves (left panels) from control (dotted line), ET-1 treated rabbits (2 ng/kg and 10 ng/kg, solid line) and histograms (right panels) for baroreflex gain (b/min/mmHg) from control (open bars) and ET-1 treated rabbits (2 ng/kg and 10 ng/kg, filled bars)**. Resting points are indicated by open circle (control) or square symbols (Et-1 Treated). Error bars are SEM, indicating variation between animals. ^*^P < 0.05.

It is possible that the interaction between endothelin and clonidine observed by Gulati and colleague is a functional antagonism rather than a pharmacological interaction. Endothelin-1 has been established as a very potent vasoconstrictor (Yanagisawa et al., 1988; Huggins et al., 1993). However, when administered at low to moderate doses, endothelin does not cause changes in blood pressure because the increase in vascular tone is masked by a baroreflex-mediated reduction of sympathetic vasomotor tone. In this case the normal sympathoinhibition produced by clonidine would not be observed and a physiological antagonism has occurred. This mechanism would not, however, explain the blockade of bradycardia by endothelin. The bradycardia induced by clonidine is due to cardiac sympathetic inhibition as well as increased vagal tone due to a facilitation of the vagal component of the baroreceptor-heart rate reflex (Badoer et al., 1983). One possibility is that the normal resetting of the baroreceptor-heart rate reflex, that permits the bradycardia to occur in the presence of a fall in blood pressure, has not taken place. Intracisternal administration of endothelin-1 produces a facilitation of the vagal component of the baroreceptor-heart rate reflex in conscious normotensive and hypertensive rats (Itoh and van den Buuse, 1991; van den Buuse and Itoh, 1993). However, in the present study we demonstrated a reduction of the gain of the baroreceptor-heart rate reflex produced by intracisternal endothelin-1 (Figure 4). This illustrates that there could be important species differences between rabbits and rats. Indeed, this also applies to the effects of clonidine. One of the most obvious differences is the relative lack of a pressor effect of intravenously injected clonidine in conscious rabbits. In rabbits, the pressor response is very transient and maximal at usually around 30 s after injection. The preponderance of the central depressor effect soon takes over and by 5 min after injection it is close to its maximum (Figure 2). By contrast, in rats the hypertensive effect following systemic injection of clonidine is much more prolonged. A further difference is that in rats there appears to be a pronounced central pressor action of clonidine, which is not observed in rabbits (Head and de Jong, 1986; Kawasaki et al., 1992).

Whatever the mechanism behind the possible interaction of endothelin-1 and clonidine in the studies from Gulati (1992), Gulati and Srimal (1993), and Lavhale et al. (2010), we failed to find any significant effect of pretreatment with endothelin-1 on the hypotensive and bradycardic action of clonidine in conscious rabbits. The dose of endothelin-1 and the pretreatment time which we used in our studies were similar to those used in the rat studies. Higher central doses of endothelin were not used in our study, as they may induce systemic or cerebral vasoconstriction and ischaemia, which could lead to direct or reflex increases in blood pressure, respectively (Macrae et al., 1991; van den Buuse, 1997). Indeed, higher doses of systemically injected endothelin-1 caused a pronounced rise in blood pressure in the present experiments. Moreover, the intracisternal injection of 50 ng/kg, but not 10 ng/kg, in the present experiments produced hypertension in one rabbit and a moderate increase in blood pressure in two others, suggesting that this dose was at the pressor threshold.

In our experiments we used conscious rabbits, while urethane-anaesthetized rats were used in the previous studies. In rabbits the doses of clonidine are close to the maximal doses for intravenous and intracisternal routes of administration and represent appropriate doses for the examination of an interaction with endothelin (Head et al., 1983). Conscious animals are preferable over anaesthetized animals as anaesthesia may alter cardiovascular reflexes and centrally-mediated responses and may affect pharmacokinetics of injected drugs, such that they are cleared or metabolized at a different rate. It cannot be excluded that the results obtained by Gulati (1992), Gulati and Srimal (1993) and Lavhale et al. (2010) can only be reproduced in anaesthetized animals.

In conclusion, endothelin-1 is an effective pressor agent in conscious rabbits. However, while previous studies in anaesthetized rats have shown that endothelins may interact with the central antihypertensive action of clonidine, at subpressor doses of endothelin we did not find evidence for such an interaction in conscious rabbits.

## Author contributions

KL, MV, GH contributed to the analysis of the data and writing of the manuscript.

### Conflict of interest statement

The authors declare that the research was conducted in the absence of any commercial or financial relationships that could be construed as a potential conflict of interest.
